# Influence of Ion Source Geometry on the Repeatability of Topographically Guided LAESI-MSI

**DOI:** 10.1021/jasms.1c00262

**Published:** 2022-01-12

**Authors:** Benjamin Bartels, Aleš Svatoš

**Affiliations:** Max Planck Institute for Chemical Ecology, 07745 Jena, Germany

## Abstract

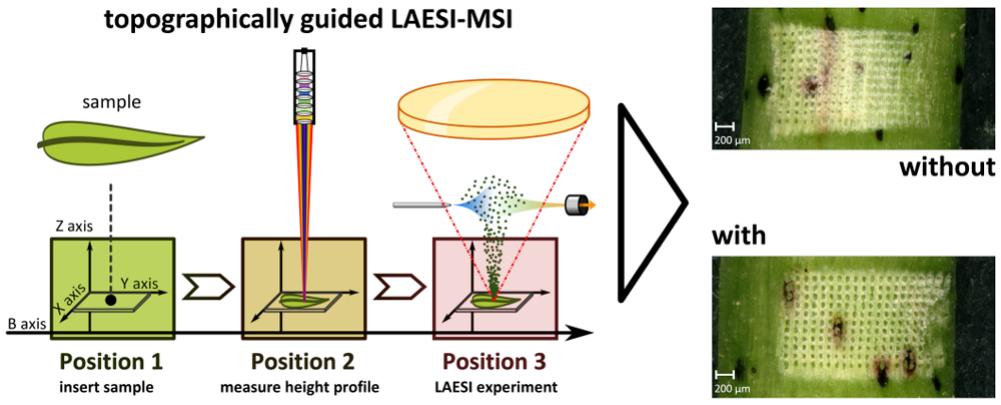

Spatially resolving the relative distribution of analyte molecules in biological matter holds great promise in the life sciences. Mass spectrometry imaging (MSI) is a technique that can provide such spatial resolution but remains underused in fields such as chemical ecology, as traditional MSI sample preparation is often chemically or morphologically invasive. Laser ablation electrospray ionization (LAESI)-MSI is a variation of MSI particularly well-suited for situations where chemical sample preparation is too invasive but provides new challenges related to the repeatability of measurement outcomes. We assess the repeatability of LAESI-MSI by sampling a droplet of [ring-^13^C_6_]l-phenylalanine with known concentration and expressing the resulting variability as a coefficient of variation, *c*_v_. In doing so, we entirely eliminate variability caused by surface morphology or underlying true sample gradients. We determine the limit of detection (LOD) for^13^C_6_-Phe by sampling from droplets with successively decreasing but known concentration. We assess the influence of source geometry on the LOD and repeatability by performing these experiments using four distinct variations of sources: one commercial and three custom-built ones. Finally, we extend our study to leaf and stem samples *Arabidopsis thaliana* and *Gossypium hirsutum*. We overcome the challenges of LAESI associated with three-dimensional surface morphology by relying on work previously published. Our measurements on both controlled standard and realistic samples give strong evidence that LAESI-MSI’s repeatability in current implementations is insufficient for MSI in chemical ecology.

## Introduction

Mass spectrometry imaging (MSI) is a rapidly growing field with an equally fast growing number of applications in lipidomics,^1,2^ proteomics,^3,4^ biotyping,^5−7^ and medical research.^8−10^ The workhorse ion sources at the forefront of this development, matrix-assisted laser desorption/ionization (MALDI)^11^ and desorption electrospray ionization (DESI),^12^ have a proven record of reliability. They do, however, impose strict requirements on sample preparation and the sample’s surface morphology.

For example, the sample surface is ideally as flat as possible to guarantee uniform analysis. Not every surface of analytical interest is flat, however, especially so in nonmedical life sciences. How to achieve uniform surface analysis in spite of nonflat surfaces has been the topic of much discussion in recent years.^13−16^

MALDI additionally requires the sample to be coated in a matrix compound prior to analysis, which may alter the chemical state of the sample prior to the actual analysis. MALDI and DESI are therefore avoided in scientific fields where any form of extensive sample preparation could be detrimental to the success of an experiment. One of these fields is chemical ecology, where the molecular composition of a sample at a defined point in time is of crucial interest. In this context, altered chemical states may lead to the wrong conclusions.

Laser ablation electrospray ionization (LAESI) is a potential alternative to MALDI. In LAESI, ionization is facilitated by an electrospray (ESI), reducing the need for sample preparation greatly.^17^ Although LAESI has been applied successfully to various samples over the past decade,^18−21^ its use in MSI continues to be infrequent.

In our experiments on *Arabidopsis thaliana* and *Gossypium hirsutum*, the measured intensity values exhibited an unexpected pixel-to-pixel variability. This observation had several conceivable explanations, which we combined in three experimentally assessable groups: first, the unexpected variability resulted directly from biology, that is, unexpected differences in local metabolite concentrations, water content, and/or tensile strength of the tissue in question; second, the electrospray providing the ionization capabilities of the LAESI ion source was not stable during the MSI experiments; or third, the ion yield of the ionization step was at least partially subject to randomness. We hypothesize that, in LAESI, ion yield is responsible for the variability because the ablation, and ESI plumes do not always interact equally.

In the following, we present the results of our investigation into the matter and discuss why the experimental evidence suggests explanation three to be the answer. To support our argument, we determined the repeatability of three custom-built and a commercial LAESI ion source by repeatedly sampling the same concentration of ^13^C_6_-Phe. Here, we also include the effects of normalization.

## Methods

### Custom-Built LAESI Ion Sources

For this work, three distinct variations on the LAESI ion source concept were custom-built, based on a prototype LAESI ion source with topographically guided laser ablation, as described earlier.^15^ All three ion sources used the same ESI capillary (part no. 700000341, Waters, Milford, MA, USA) and solvent delivery system, a LC-20AD Prominence binary pump (Shimadzu, Kyoto, Japan) together with a 1100 series degassing unit (Agilent, Santa Clara, CA, USA), the same four-dimensional sample manipulation system comprising two MZS50/M-Z8, a MZS25/M-Z8, and a DDS220/M linear translation stage (Thorlabs, Newton, NJ, USA). The optical pathway for all three ion sources consisted of four turning mirrors (PF10-03-M01, Thorlabs) in kinematic mounts (KCB1/M and KCB1C/M, Thorlabs), a wire-grid polarizer (WP25M-UB, Thorlabs) in a rotating mount (CRM1/M, Thorlabs) for laser energy attenuation, a 2 mm pinhole (custom-built, Thorlabs), and a 1:10 telescope (LA5315-E and LA5714-E, Thorlabs) to widen the beam in front of the focusing lens. The focusing lens was an aspheric ZnSe lens with either 50 mm (AL72550-E, Thorlabs) or 25 mm (AL72525-E, Thorlabs), depending on the source geometry. In the case of the 25 mm focusing lens, an additional turning mirror (MRA25-M01) was necessary. The smallest achievable ablation mark diameters on thermoactive paper and the living tissue of *Gossypium hirsutum* was 30 μm on average. In MSI experiments, the ablation mark diameter was maximized to fit the lateral resolution. We avoided oversampling through profilometry and controlled defocusing, as described in a previous publication.^15^ In all setups, a stable electrospray was achieved with 1% (v/v) formic acid and 100 ng mL^–1^ leucine enkephalin in a 1:1:1:1 (v/v/v/v) mixture of water, methanol, isopropyl alchol, and acetonitrile, at a flow rate of 1.2 μL min^–1^ and a capillary voltage of 3.5 to 4.1 kV and from −3.6 to −4.2 kV for positive and negative ion mode, respectively. The same Synapt HDMS TOF-MS (Waters) was used with all three LAESI ion source variations.

**Figure 1:**
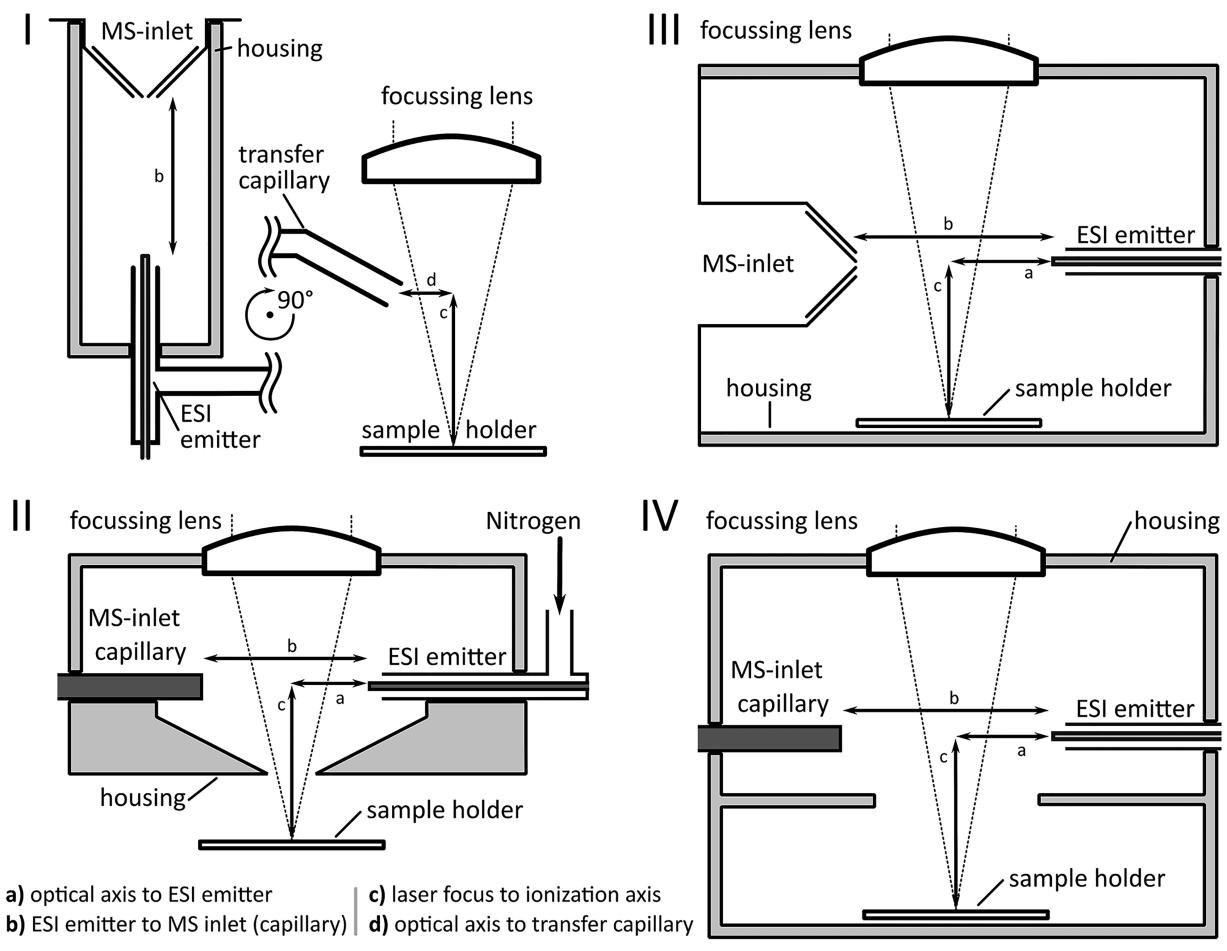
Schematic representations of the investigated LAESI ion source geometries, namely, coaxial ionization (I), the ionization chamber (II), and classic LAESI geometries (III), as well as the commercial DP-1000 LAESI ion source (IV).

Each variation on the LAESI ion source concept, schematic representations of which are shown in Figure 1, was designed to investigate a particular aspect of the ionization step in the LAESI process. The first variation, henceforth referred to as the classic LAESI geometry, was designed to resemble as closely as possible the geometry described in the original LAESI publication,^17^ while retaining the optics and sample manipulation system described above. To achieve this resemblance, the ESI emitter was placed directly in front of the MS inlet at a distance of 10 mm and 14 mm above the focal point of the AL72550-E focusing lens. The laser axis intersected the axis between MS inlet and ESI capillary 6.0 mm in front of the ESI emitter.

In the second variation, in the following referred to as ionization chamber geometry, the interaction of the ablation and ESI plume took place in a chamber rather than in front of the MS inlet. The transport of ions to the mass spectrometer was facilitated by a steel capillary. Inside the ionization chamber, the ESI emitter was placed 10 mm in front of the steel capillary tube and 13 mm above the focal point of the AL72525-E lens. The laser axis intersected the axis between the steel and ESI capillary tubes 6.5 mm in front of the ESI emitter.

In the third variation, henceforth referred to as the coaxial ionization geometry, the laser ablation step and the electrospray ionization were spatially separated. The ESI emitter was positioned 10 mm in front of the MS inlet within an airtight housing. Stainless-steel capillary tubes (U-139 and U-145, IDEX Corporation, Lake Forest, IL, USA) and a P-714 PEEK tee-connector (IDEX) were used to construct an ablation plume transfer capillary tube and a sheath-gas capillary tube; these were connected to allow ablated sample material to be incorporated into the gas stream around the ESI (the gas stream made a sheath of the atmosphere sucked in by the vacuum of the MS instrument). The opening of the capillary tube in which the ablation plume transfer took place was positioned 13 mm above the focal point of the AL72525-E lens and 3.0 mm off the optical axis.

All experiments were conducted with a commercially available LAESI DP-1000 ion source (Protea Bioscience, Morgantown, WV, USA) as a control against any construction bias of our custom-built ion sources. In all experiments, the DP-1000 was connected to the same XEVO qTOF instrument (Waters). In the DP-1000 LAESI ion source, the laser axis intersected the axis between MS inlet capillary and ESI capillary, which were 11 mm apart, 6.0 mm in front of the ESI emitter and roughly 14 mm above the focal point of the laser.

### LAESI-MSI with Topographically Guided Laser Ablation

All MSI experiments presented here were conducted with the classic LAESI ion source geometry that made use of our previously published approach^15^ to use profilometric data to move the sample up and down to compensate for a nonflat surface morphology. Spatial context was provided by recording laser activity as a squared voltage signal in the analog channel of the Synapt Instrument. An Arduino UNO microcontroller, expanded upon with a Screw Shield 1.0 (Conrad Electronic SE, Hirschau, Germany), was positioned between the Q-switch output of the laser and the analog input channel of the MS instrument to elongate and transform the squared signal emitted by the laser. Assignment of mass spectra and conversion of the raw data to imzML data sets^22^ was done in the R software environment (v3.6.0., R Development Core Team, 2019), with the help of the MALDIquant package.^23,24^

For experiments on *A. thaliana*, the fifth leaf of a plant was separated from the rosette and fixed with the abaxial side up to a microscope slide with double-sided adhesive tape. The height profile of a region of interest (ROI), usually 10 by 5 mm, was measured by the integrated profilometer at a lateral resolution of 200 μm. Based on the height profile acquired, the ROI was then sampled at the same lateral resolution, with the compensatory sample stage movement along the vertical axis to account for the nonflat surface morphology of the leaf. Laser ablation took place with 20 laser pulses per position, at a repetition rate of 20 Hz and an laser energy of 35(1) μJ per pulse, to account for the nonflat surface morphology of the leaf.

The experiments on *G. hirsutum* were performed on transversally cut stems that were positioned flat side down on microscope glass slides without further fixation. ROIs measured 2 by 1 mm and were sampled at a lateral resolution of 100 μm with 20 laser pulses at 20 Hz repetition rate and an energy of 71(2) μJ per pulse after the corresponding height profile was measured for topographical guidance. Optical images were taken pre- and postexperiment with a VHX-5000 digital stereomicroscope in conjunction with a VH-Z20R objective and an OP-87429 polarization filter (Keyence, Osaka, Japan).

### Determination of the ^13^C_6_-Phe Limit of Detection (LOD)

Standard solutions of 1, 10, 50, 100, and 500 μg mL^–1^ [ring-^13^C_6_]l-phenylalanine concentration were prepared from a stock solution (Cambridge Isotope Laboratories Inc., Tewksbury, MA, USA). From each of these solutions, three droplets of 2 μL volume were sampled, one at a time, with 11 bursts of five laser pulses at 20 Hz repetition rate and a laser energy of approximately 75 μJ per pulse. A droplet of 20 μL volume had to be provided in the case of the DP-1000 ion source because the increased beam diameter led to an increased amount of sample being ablated. Laser ablation in the DP-1000 ion source took place at 10 Hz and 1 mJ laser energy per pulse. For the purposes of evaluation, the intensity value of the *m*/*z* 126.11 fragment was considered instead of the response of the protonated molecule. A signal-to-noise (S/N) threshold of 4 was used to determine the LOD.

Statistical analysis was performed in the R software environment. The signal variation, which increased with increasing concentrations, made it necessary to describe the influence of the concentration on the signal intensity with a quadratic regression model. The variance heterogeneity was accounted for using a generalized least-squares method (function “gls” of the “nlme” library^25^) employing the “varPower” or the “varExp” structure to weight the residual variances. The variance structure was determined by the model and a likelihood ratio test, as well as by selecting the model with the smallest AIC.^26^

### Determination of the Coefficient of Variation

The coefficient of variation, *c*_v_, of all four LAESI ion sources was determined experimentally by sampling a droplet of the ^13^C_6_-Phe standard of known concentration multiple times in short succession. To this end, a 2 μL droplet of ^13^C_6_-Phe standard was sampled with 11 bursts of five laser pulses at 20 Hz repetition rate, a laser energy of approximately 75 μJ per pulse, with a 2 s break between each sampling. In the LAESI DP-1000 ion source, a 20 μL droplet was sampled at a 10 Hz repetition rate and a laser energy of approximately 1 mJ per pulse. The concentration of ^13^C_6_-Phe in the droplet presented was 100 μg mL^–1^ in the ionization chamber and classic LAESI geometry as well as in the DP-1000 ion source. For the sample involving coaxial ionization geometry, the concentration was increased to 500 μg mL^–1^ to ensure a sufficient response. The experiment was repeated ten times in each ionization source. The intensity value of the *m*/*z* 126.11 fragment was measured for each source. The coefficient of variation, *c*_v_, was consistently calculated as the ratio between standard deviation (σ) and mean intensity (*x̅*) of *m*/*z* 126.11. The experiments to determine the *c*_v_ were also performed without presenting droplets to assess the influence of ablation plume expansion on electrospray stability.

### ESI Current Measurements

The electric current drawn by the electrospray was measured during the experiments determining the *c*_v_. The custom-built ESI current meter (IOCB, Prague, Czechia) was situated between the power supply of the MS instrument and the ESI emitter and translated the drawn current into a recordable voltage signal. A NI-6008 DAQ device (National Instruments, Austin, TX, USA) was used to record this voltage signal; a second voltage signal indicated the laser activity of the laser’s power supply unit.

## Results and Discussion

### LAESI-MSI with Topographically Guided Laser Ablation

**Figure 2:**
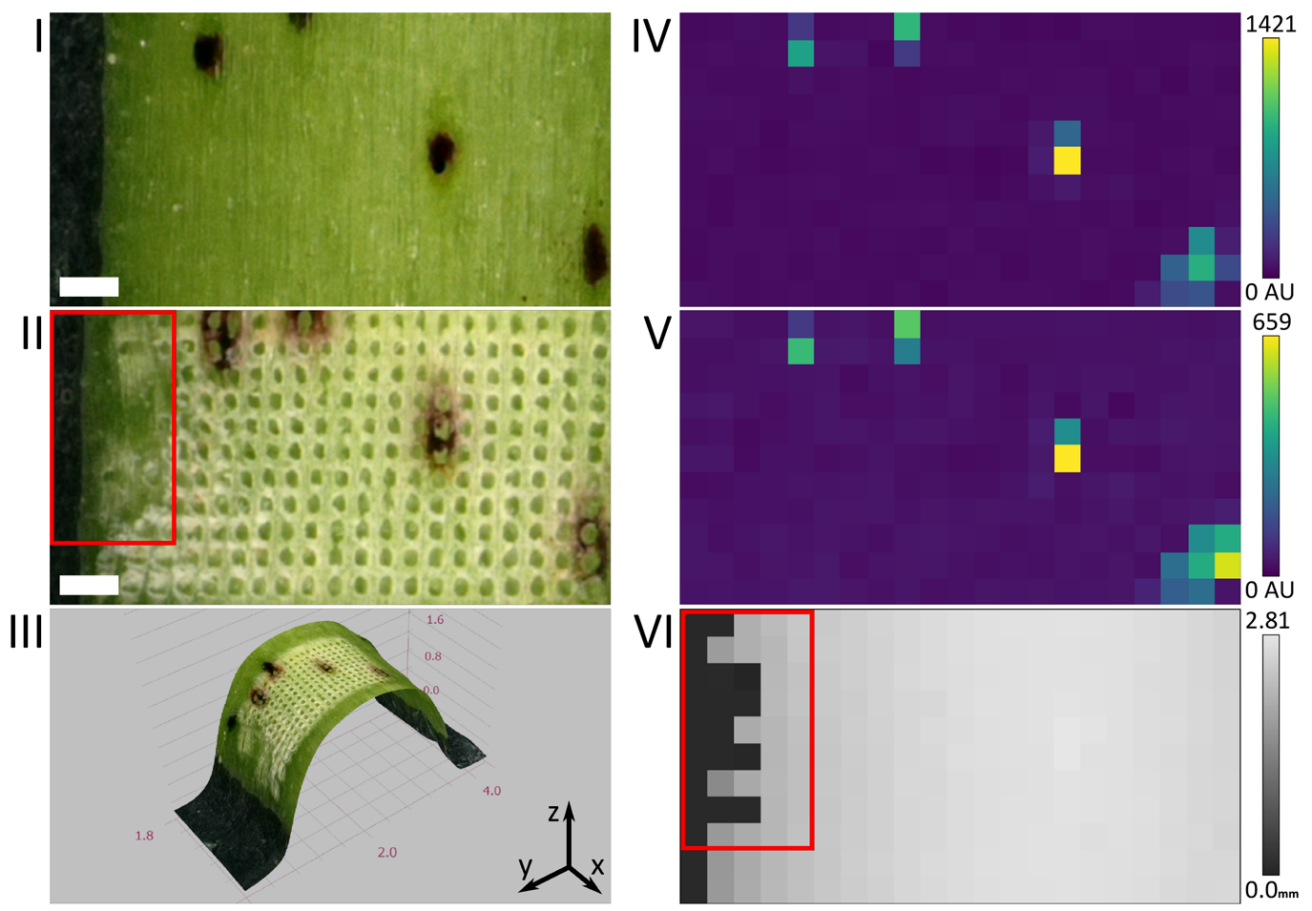
(I, II) Optical image of a *G. hirsutum* stem at 50-fold magnification, pre- and post-LAESI-MSI, respectively. (III) Three-dimensional rendering of the ROI, based on an acquired *Z*-stack of optical images taken post-LAESI-MSI. The spatial distributions of *m*/*z* 273.08 (IV) and 409.20 (V) were acquired at 100 μm lateral resolution in our custom-built ion source, configured in the classic LAESI geometry and putatively assigned to the [M – H]^⊖^ of hemigossypolone and heliocide H1-4, respectively. The topographic map (VI) was acquired by profilometry prior to LAESI-MSI and visualizes, in gray scale, the height values for each sampling position of the LAESI-MSI. The red marking indicates an area where surface curvature interfered with the profilometry and thus laser ablation. All scale bars represent a distance of 200 μm.

We performed LAESI-MSI on the surface of *G. hirsutum* stems for the first time. The application of topographical guidance was essential in compensating for the surface curvature of the stems and therefore to ensure the consistent laser ablation necessary for LAESI-MSI. Figure 2 shows results of LAESI-MSI on a representative stem sample, obtained with our custom LAESI ion source configured in the classic LAESI geometry. As observable in panel II, laser ablation was consistent except for the left most rows, marked in red. At the marked sampling positions, laser ablation failed because the curvature of the stem’s surface was too high for reliable profilometry. A three-dimensional rendering of the ROI (panel III), based on an acquired *Z*-stack of optical images taken postablation, illustrates the correlation between failed laser ablation and surface curvature optically. Similarly, the topographic map of the ROI, shown in panel IV, features irregularities in the sampling positions that correlate with the unablated sampling positions (red marking) visible in the optical images.

The spatial distributions of *m*/*z* 273.08 and 409.20 (panels IV and V), putatively assigned to hemigossypolone and heliocide H1-4, respectively, exhibit the expected binary and highly localized distribution within the black pigment glands.^27^ Representative mass spectra for black pigment glands as well as green tissue can be found in the Supporting Information (Figure S2).

**Figure 3:**
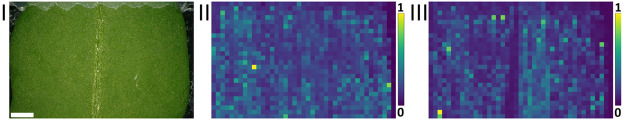
(I) Optical image of an *A. thaliana* leaf taken pre LAESI-MSI, as well as the spatial distributions of *m*/*z* 447.05 (II) and 223.06 (III), assigned putatively to the [M – H]^⊖^ of glucobrassicin and sinapic acid, respectively. The visualized intensity values were acquired at 200 μm lateral resolution in our custom-built ion source configured in the classic LAESI geometry and TIC-normalized. The scale bar represents a distance of 500 μm.

As a plant of the Brassicaceae family, *A. thaliana* produces glucosinolates as defensive metabolites.^28^ These are distributed evenly, or with gradual changes, in the plant’s leaves,^29,30^ in contrast to the binary distribution of compounds present in the black pigment glands of *G. hirsutum*. We expected glucobrassicin to have the most homogeneous distribution of the multiple glucosinolate compounds present in *A. thaliana* leaves. Any measured distribution was therefore expected to exhibit low pixel-to-pixel variability. Representative results from LAESI-MSI experiments on *A. thaliana* leaves are shown in Figure 3. Panels II and III show the spatial distributions of *m*/*z* 447.05 and 223.06, putatively assigned to glucobrassicin and sinapic acid, respectively. Although the distributions shown in Figure 3 visualize TIC-normalized intensity values, to account for small fluctuations in the overall ion yield of the electrospray, the high pixel-to-pixel variation in the measured intensity values masks potential smaller changes in intensity that would indicate gradual changes in metabolite distribution. The difference in distribution of glucobrassicin (II) and sinapic acid (III) is the absence of the latter in the midrib of the leaf, suggesting that stark contrasts in metabolite distribution were still picked up. Representative mass spectra acquired during LAESI-MSI on *A. thaliana* leaves can be found in the Supporting Information (Figure S3). The surprisingly high pixel-to-pixel variability sparked our interest in the repeatability achievable with LAESI and our custom-built ion sources.

### Limit of Detection and Repeatability

Measurements of the ^13^C_6_-Phe LODs of all three custom-built LAESI ion source geometries and the LAESI DP-1000 ion source were used to estimate how LAESI ion source geometry might influence sensitivity under otherwise equal conditions. Table 1 lists the determined LOD values, based on the quadratic regression modeling and an S/N threshold of 4. Figure S1 in the Supporting Information shows the collected data points for all four ion sources tested. Transporting the ablated sample as electrically neutral particles, as is the case with the coaxial source geometry, seems to influence the LOD negatively. In all cases, the observed signal variation increased significantly with the concentration of ^13^C_6_-Phe, negatively impacting the quality of the regression modeling, despite appropriate variance correction and reflected in the confidence intervals. The measured LOD values were therefore interpreted as estimates and not taken at face value.

**Table 1:** Calculated ^13^C_6_-Phe Limit of Detection Values, Including Confidence Intervals (*p* = 0.05) for All Four Ion Source Geometries Tested and the Achieved Laser Focus and Sample Volume

ion source geometry	LOD (μg mL^–1^)	laser focus (μm)	*V* (μL, sample)
ionization chamber geometry	0.86(48)	100	2
classic LAESI geometry	2.5(25)	100	2
coaxial source geometry	32(30)	100	2
DP-1000 LAESI source	4.3(49)	300	20

**Figure 4:**
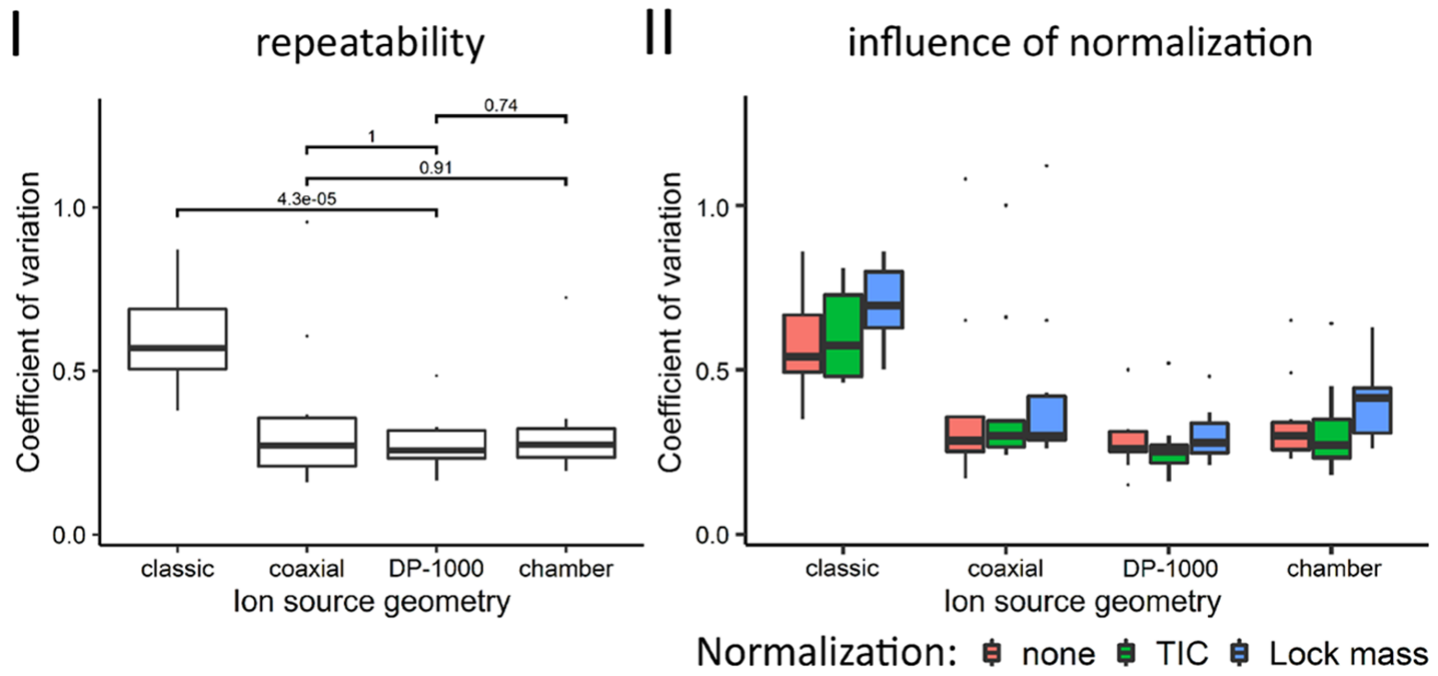
(I) Repeatability of sampling of ^13^C_6_-Phe standard droplets (*n* = 10) in the classic LAESI, coaxial ionization, and ionization chamber geometry, as well as the DP-1000 ion source, expressed as the coefficients of variation of the measured intensity values. The numbers above the brackets denote the corresponding *p*-value of a pairwise *t* test. Normalization by TIC or lock mass (II) has no significant influence on the coefficient of variation (ANOVA, *p* = 0.05).

The coefficient of variation, *c*_v_, was the parameter of choice to evaluate the repeatability. Figure 4, panel I displays the *c*_v_ calculated from the experimental data acquired by ion source geometry. Measurements using the classic LAESI geometry are significantly less repeatable than experiments with the other ion source geometries. The average coefficients calculated from the raw data are 0.61, 0.32, 0.28, and 0.35 for the classic LAESI, ionization chamber, DP-1000, and coaxial ionization geometries, respectively. No change resulted when the measured ^13^C_6_-Phe intensity values, either by TIC or leucine enkephalin, were normalized as shown in Figure 4, panel II (ANOVA, *p* = 0.05). In general, a *c*_v_ of 0.25 (calculated from data from 10 experiments) represents a situation in which the standard deviation equals a quarter of the mean. Under these circumstances, a signal response needs to either double or be halved to be considered significantly different. Although *c*_v_ is derived from intensity values, the same arguments holds true when considering concentrations due to the relative and dimensionless nature of *c*_v_.

### ESI Stability

**Figure 5:**
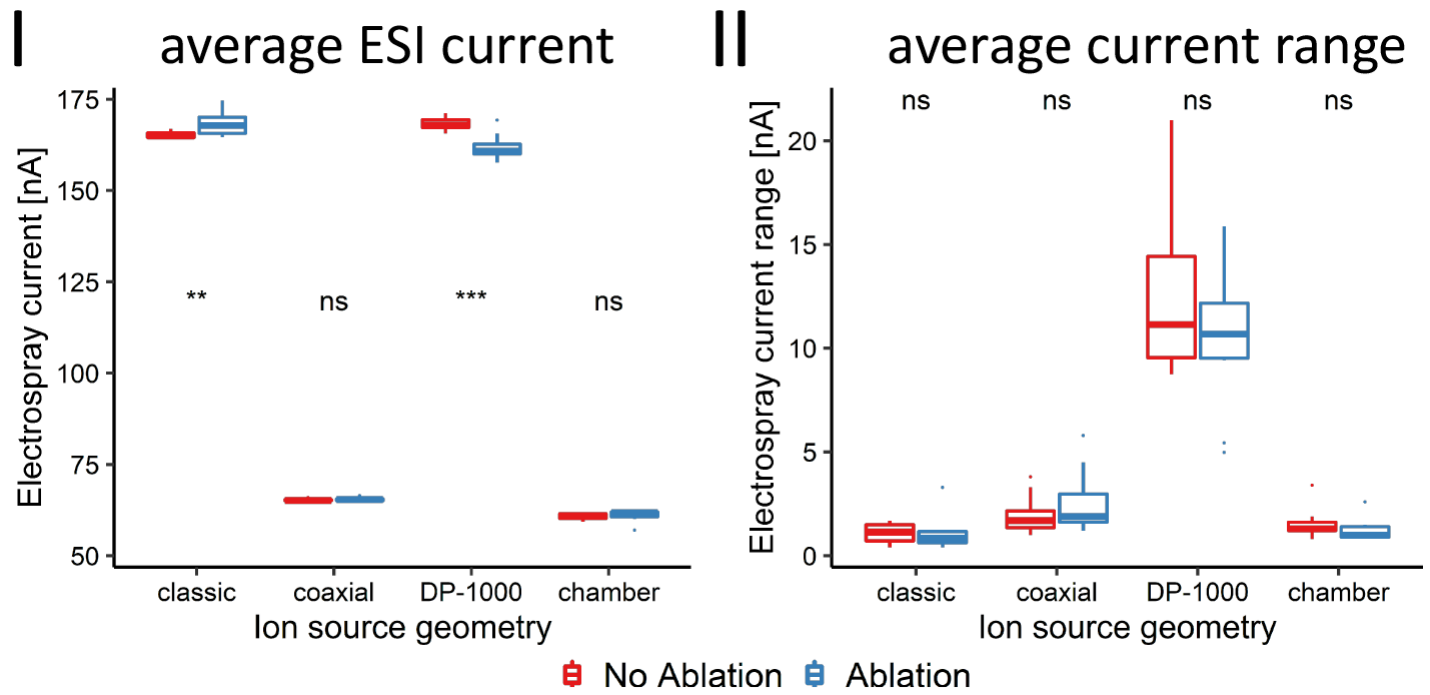
(I) Average electric current drawn by the electrospray during the repeatability (blue) and the control experiments with no liquid standard present in the source (red), per investigated ion source geometry. In (II), the corresponding range of electric current drawn is shown in the same color scheme as that used for (I).

In Figure 5, panels I and II, the amount of average ESI current and the range of ESI current drawn are shown for each ion source geometry, respectively. In general, the average amount of ESI current drawn by the classic LAESI and the DP-1000 source geometry was higher than that drawn by the other two spatially more confined ion source geometries. Furthermore, the amount of current drawn in both the classic LAESI and the DP-1000 source geometry differed significantly (*p* < 0.01 and *p* < 0.001, respectively) between the *c*_v_ determination and control experiments (no sample being presented); however, the observed trends are reversed. The classic LAESI geometry drew more and the DP-1000 source drew less ESI current in the *c*_v_ determination experiments, compared to the control experiments. No significant differences were observed in the other two ion source geometries. In addition to the average ESI current, the range of currents drawn was considered a suitable parameter to assess the stability of ESI over the course of the experiments. No significant differences were observed for any ion source geometry between the experiments with ablation taking place and the control experiments. It was therefore concluded that the ablation events did not influence the long-term stability of the ESI.

### Discussion

MSI is a relatively quantitative methodology. Experimental parameters must remain constant over the course of an experiment if results are to be comparable. We were therefore concerned with the repeatability of our custom-built LAESI ion sources after observing the unexpected pixel-to-pixel variability in the experiments on *A. thaliana*.

There are several conceivable explanations for the observed variability, which fall into three experimentally assessable groups. First, the variability of measured intensity values was due to naturally occurring differences of biological parameters, such as local metabolite concentrations, water content, and tensile strength of the sampled tissue. Second, the electrospray providing the ionization capabilities of the LAESI ion source was not stable (the consequences of which having been studied previously^31^). Third, the ion yield of the ionization step was partially subject to randomness, resulting from an inconsistent interaction between the ablation and ESI plumes. To date, the third possibility has only rarely been considered.

Although Stopka et al.^20^ reported biologically derived differences in local analyte concentrations being measured in a fiber-based LAESI ion source, their measurements were obtained from ablating a single cell. In the MSI experiments we reported here, the average ablation mark removed a volume far larger than a single cell. Even when significant differences in local concentrations may have been present, any variation originating from different metabolite concentrations in single cells was inevitably reduced through averaging effects.

Similarly, electrospray instability is not supported as an explanation for the observed variability by our data either. In our experiments, the range of current drawn by the electrosprays of all investigated ion sources remained approximately similar whether or not laser ablation took place, suggesting that the ablation event does not lead to a destabilization of the ESI plume. A randomness in the analytical volume created by the overlapping ablation and ESI plumes, however, could cause a fluctuating ion yield. Measured intensity values might then vary on a pixel-by-pixel basis, depending on how well the ESI and the ablation plume overlap.

The unique possibility of LAESI to directly sample liquids made it possible to eliminate variability caused by biological parameters entirely. The distribution of a (solvated) standard within a solution can safely be assumed to be homogeneous, unlike the distribution of standards in homogenates and sprayed-on targets used in most experiments with MALDI or DESI sources. Sampling a droplet of a standard solution repeatedly therefore truly means sampling the same concentration repeatedly. The inclusion of the commercially available LAESI ion source controlled against a possible bias introduced by the in-house construction of the three other ion source geometries.

In the end, the lack of repeatability observed here would be of little consequence in any kind of exploratory profiling experiments. It limits, however, the usefulness of LAESI as an ionization technique for MSI experiments to applications with very distinct spatial distributions of metabolites. Studies of the transport of neutral particles in remote LAESI geometries,^32^ such as that of Dolatmoradi et al.,^33^ are therefore important for the ongoing development of the LAESI method. The transmission geometry of the optical system applied in that study, however, is unsuitable for applications on samples with a thickness beyond that of a thin section and with a three-dimensional surface topography, such as stems and whole leaves.

## Conclusion

As a technique, LAESI offers a unique approach to ionization; it holds great promise for application-rich fields with delicate samples, such as chemical ecology, especially when combining profilometry of the sample’s surface and topographically guided laser ablation. More work on the interaction between ablated sample material and the ESI plume will be necessary, however, before its advantages are evident in routine MSI experiments.

## Supporting Information Available

In-depth explanation of how to estimate the minimal difference in local metabolite concentration (relatively) based on a previously determined coefficient of variation; list of the determined intensity-based as well as the calculated concentration-based coefficient of variation for each tested ion source (Table S1); regression parameters of the quadratic regression performed to determine the LOD values of each tested ion source for ^13^C_6_-Phe (Table S2); all data points collected in the LOD experiments for all four ion sources tested (Figure S1); representative mass spectra acquired during the LAESI-MSI experiments on *G. hirsutum* and *A. thaliana*, respectively (Figures S2 and S3) (PDF)
