# Supplementary material for: Influence of Ion Source Geometry on the Repeatability of Topographically Guided LAESI-MSI
Source: J Am Soc Mass Spectrom. 2022 Jan 12;33(2):265–72. doi: 10.1021/jasms.1c00262 (PMC8815068; doi:10.1021/jasms.1c00262)
Supplement: Supplementary file 1 — js1c00262_si_001.pdf [file js1c00262_si_001.pdf]

The influence of ion source geometry on the repeatability of topographically guided LAESI-MSI

Benjamin Bartels[†,‡] and Aleš Svatoš[*,†]

†*Max Planck Institute for chemical Ecology, 07745 Jena, Germany*

‡*Current address: M4I, 6229 ER Maastricht, The Netherlands*

E-mail: svatos@ice.mpg.de

Phone: +49 (0)3641 571700

Supporting Information Available

Minimal difference in local concentration that results in a statistically different signal measured

Definition of the coefficient of variation:

$$c_v = \frac{\sigma}{\overline{x}} \tag{1}$$

Definition of a two sided t-test:

$$t = \frac{x_1 - x_2}{\sigma_1 + \sigma_2} = \frac{\Delta x}{\Sigma \sigma} \tag{2}$$

From the assumption that the standard deviation stays the same when measuring with the same ion source twice follows:

$$t = \frac{\Delta x}{2\sigma} \tag{3}$$

Solving for the difference in signal intensities $\Delta\overline{x}$ leads to:

$$\Delta x = t * 2\sigma \tag{4}$$

The standard deviation can therefore be expressed dependent on the signal intensity with formula 1, with the difference in signal intensity as a function of c_v and signal intensity in general ($\overline{x}$):

$$\Delta x = t * 2 * c_v * \overline{x} \tag{5}$$

The experimentally determined c_v are based on 10 replicates, comparing two intensities therefore results in 18 degrees of freedom. Assuming $\alpha = 0.05$, the t-quantile for a two sided test is therefore 2.101, yielding :

$$\Delta x = 2.101 * 2 * 0.25 * \overline{x} = 1.0505\overline{x} \tag{6}$$

given a $c_v = 0.25$. The change in signal response from sampling two different concentrations must therefore result in a 100% increase in signal response to be considered significantly different. On the basis of the quadratic model used to describe the relation between measured concentration and signal response:

$$x_c = a * c^2 + b * c + d \tag{7}$$

where a, b, and d are the regression parameters given in Table **??**, x_c is the measured signal response, and c is the $^{13}C_6$ - Phe concentration of the standard. The following formula can be applied to express measured intensity values as a concentration estimate:

$$c_{(x)} = -\frac{b}{2a} \pm \sqrt{\frac{b^2}{4a^2} - \frac{d}{a} + \frac{x}{a}} \tag{8}$$

A concentration-based coefficient of variation ($c_{v(c)}$) can therefore be estimated for every

ion source geometry. The calculated $c_{v(c)}$ are listed in Table ??. The coefficient of variation derived from the originally measured intensity values ($c_{v(x)}$) and coefficient of variation based on concentration estimates of the regression model ($c_{v(c)}$) follow the same trend and are of comparable value. It seems therefore reasonable to transfer the conclusion of formula 6 to the differentiation of two concentrations:

$$\Delta c = 2.101 * 2 * 0.25 * \bar{c} = 1.0505\bar{c} \tag{9}$$

Table S1: Coefficients of variation determined for all for ion source geometries from intensity values ($c_{v(x)}$) and concentration estimates ($c_{v(c)}$).

ion source geometry	$c_{v(x)}$	$c_{v(c)}$
ionization chamber	0.32	0.31
classic LAESI	0.61	0.49
DP-1000 LAESI	0.28	0.21
coaxial ionization	0.35	0.22

Table S2: Parameters determined for quadratic regression fitting of the LOD data sets. General regression formula: $x_c = ac^2 + bc + d$ with c being the concentration of L-phenylalanin. Values in brackets denote p-values.

ion source geometry	a	b	d	weighting
ionization chamber	-0.0013 (0.071)	2.423 (0.001)	-0.0836 (0.803)	'varPower'
classic LAESI	0.0043 (0.068)	2.961 (0.001)	2.727 (0.030)	'varPower'
DP-1000 LAESI	0.0022 (0.019)	1.418 (0.001)	17.808 (0.004)	'varPower'
coaxial ionization	0.0002 (0.014)	0.1581 (0.002)	2.706 (0.166)	'varExp'

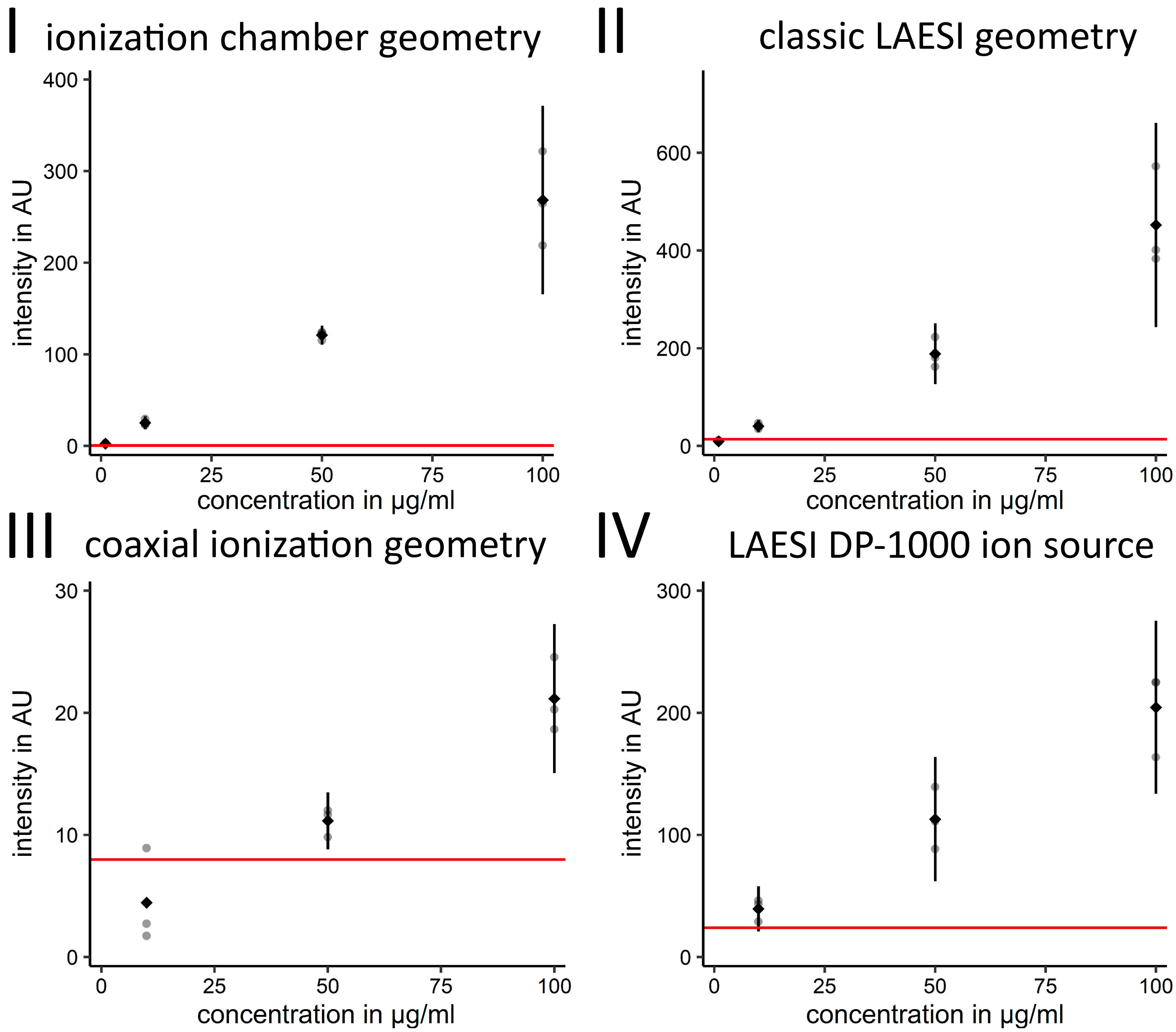

Figure S1: $^{13}C_6$ - Phe concentration versus intensity value measured with the ionization chamber (**I**), classic LAESI (**II**), coaxial ionization geometry (**III**), and DP-1000 ion source (**IV**), respectively. The red line marks the detection limit of signal/noise (4).

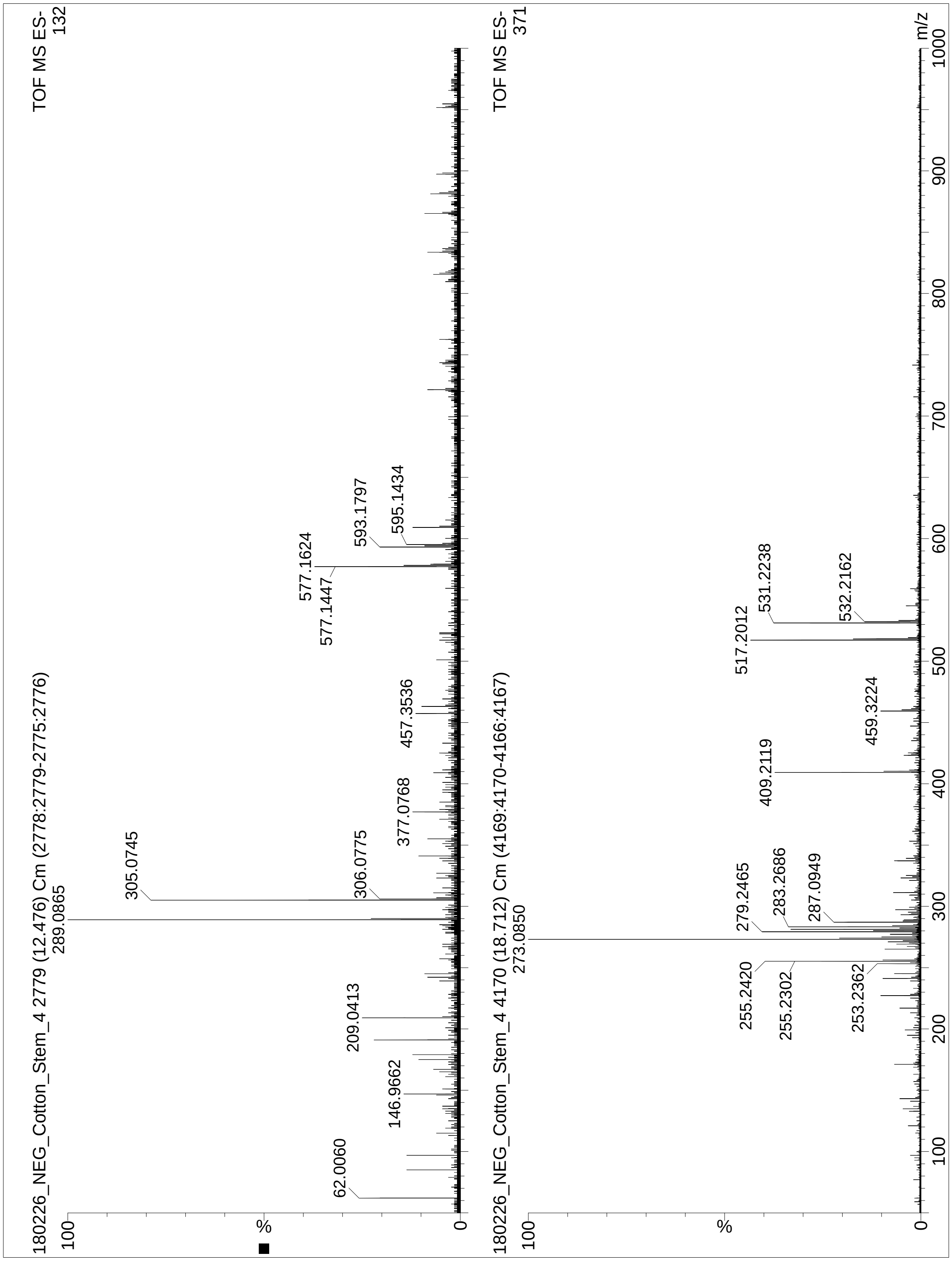

Figure S2: Representative mass spectra of green tissue (left) and pigment gland (right) from the proof-of-concept experiments on *G. hirsutum* stem, measured with the classic LAESI source geometry.

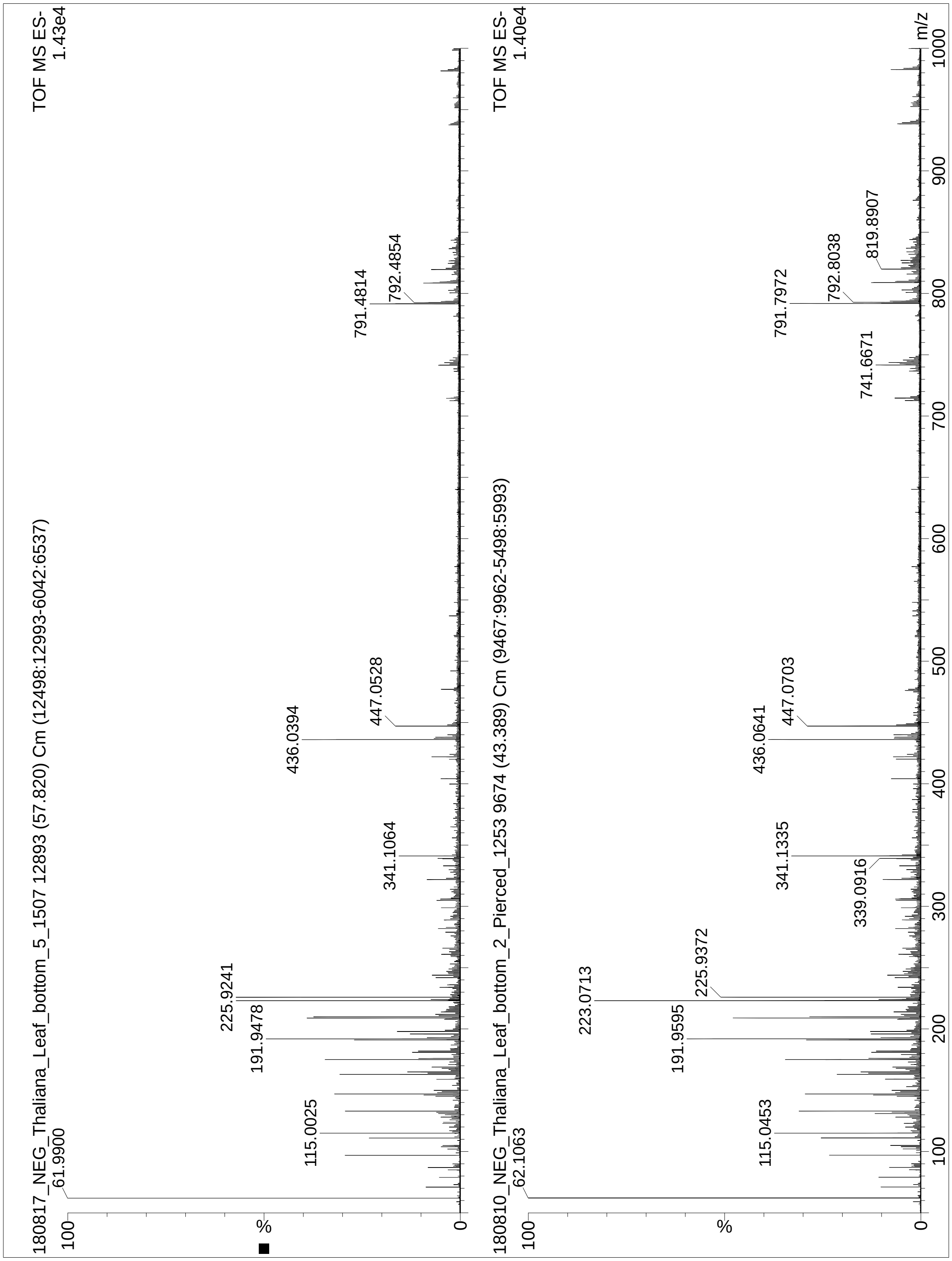

Figure S3: Representative mass spectra from *A. thaliana* leaves measured during LAESI experiments with the classic LAESI source geometry.
